# Relationship between meiotic behaviour and fertility in backcross-1 derivatives of the [(*Gossypium hirsutum* × *G. thurberi*)^2^ × *G. longicalyx*] trispecies hybrid

**DOI:** 10.3897/CompCytogen.v14i1.47231

**Published:** 2020-01-28

**Authors:** N’guessan Olivier Konan, Guy Mergeai

**Affiliations:** 1 Gembloux Agro-Bio Tech, Liège University, Tropical agriculture Unit, 2 passage des Déportés, B-5030 Gembloux, Belgium Liège University Gembloux Belgium; 2 Jean Lorougnon Guédé University, Agroforestry Unit, BP 150, Cote D’ivoire Jean Lorougnon Guédé University Daloa Cote d'Ivoire

**Keywords:** chromosome, cytogenetics, fiber fineness, *Gossypium* spp, hybrid, *in situ* hybridization, meiosis, plant breeding

## Abstract

Wild cotton species are an important source of desirable genes for genetic improvement of cultivated cotton *Gossypium hirsutum* Linnaeus, 1763. For the success of such an improvement, chromosome pairings and recombinations in hybrids are fundamental. The wild African species *G. longicalyx* Hutchinson & Lee, 1958 could be used as donor of the desirable trait of fiber fineness. Twelve BC1 plants obtained from the backcrossing of [(*G. hirsutum* × *G. thurberi* Todaro, 1877)^2^ × *G. longicalyx*] (A_h_D_h_D_1_F_1_, 2n = 4x = 52) trispecies hybrid (HTL) by *G. hirsutum* (cv. C2) (A_h_A_h_D_h_D_h_, 2n = 4x = 52) were investigated for meiotic behaviour and plant fertility. Their chromosome associations varied as follows: (2.5 to 11.5) I + (17 to 22) II + (0.31 to 1.93) III + (0.09 to 1.93) IV + (0 to 0.07) V + (0 to 0.14) VI. Their pollen fertility ranged from 4.67 to 32.10 %. Only four BC1 plants produced a few seeds through self-pollination. The remaining BC1 were totally self-sterile and usually presented the highest number of univalents. All BC1 materials produced BC2 seeds (0.44 to 6.50 seeds per backcross) with the number of seeds negatively correlated with the number of univalents (R^2^ = 0.45, P < 0.05). Most BC1 plants gave significantly finer fiber compared to the cultivated *G. hirsutum*. SSR markers showed a segregation of wild alleles among the backcross derivatives and Genomic *in situ* hybridization (GISH) revealed presence of entire chromosomes of *G. longicalyx* as well as recombinant chromosomes in the backcross derivatives. The significance and details of these results are presented and the prospects of successfully exploiting these plant materials are discussed.

## Introduction

Cotton is the most important fiber crop in the world. It belongs to the genus *Gossypium* which comprises about 53 species (Wendel and Grover 2015, Wu et al. 2018). Among them, 46 species have been assigned to eight cytologically and geographically defined diploid genome groups (A, B, C, D, E, F, G, and K) with 2n = 2x = 26 chromosomes, and 7 species have been attributed to a tetraploid genome group (AD) with 2n = 4x = 52 chromosomes (Wendel and Grover 2015; Chen et al. 2016; Wu et al. 2018). The genome sizes ranging from largest to smallest in the following order A > F > B > E > C > G > K > D (Zhang et al. 2008) and the affinity between these genomes to the A genome, based on chromosome pairings, follows slightly the same order.

Only four cotton species are cultivated, of which *G. herbaceum* Linnaeus, 1753 (A_1_ genome) and *G. arboreum* Linnaeus, 1753 (A_2_ genome) are diploid, while *G. hirsutum* Linnaeus, 1763 ((AD)_1_ genome) and *G. barbadense* Linnaeus, 1753 ((AD)_2_ genome) are tetraploid (Wendel et al. 2009, Chen et al. 2016, Ulloa et al. 2017). *G. hirsutum* is the main cultivated cotton with more than 90 % of the world production of lint (International Cotton Advisory Committee -ICAC- 2019). Except for these four cultivated types, all the other species of the genus are wild.

In cotton breeding, wild species are an important source of several desirable genes for genetic improvement of *G. hirsutum* such as fiber quality, resistance to diseases and insect pests, or tolerance to abiotic stress. The wild species *G. longicalyx* Hutchinson & Lee, 1958 (F_1_ genome) could be used as donor of the desirable traits of fiber fineness, length and strength, which are very important to textile industry (Demol et al. 1978, Ndungo et al. 1988, Nacoulima et al. 2016). *G. longicalyx* appears to be a mixed genome related to all the other cotton genomes (except D genome) and phylogenetic analysis suggests a close relationship between its F_1_ genome and the A genome (Cronn et al. 2002). In 2007, Konan et al. have created the HTL trispecies hybrid by crossing the [*G. hirsutum* ((AD)_1_ genome) × *G. thurberi* Todaro, 1877 (D_1_ genome)]^2^ hexaploid to *G. longicalyx* (F_1_ genome). This hybrid was totally self-sterile and its interspecific status was confirmed using SSR markers and cytogenetic analysis (Konan et al. 2007), but no data have been published so far concerning the meiotic behaviour and the fertility of its progeny.

In interspecific breeding programs, carrying out continuous cytological analysis is very important for plant selection because it provides information concerning the degree of meiotic irregularities, viability of gametes, chromosome pairing and genetic recombination (Lavinscky et al. 2017). For introgression of the desirable characters from the donor into the recipient, homoeologous recombinations are essential and the occurrence of bivalents and multivalents is important because they are indicative of chromosome recombination. It is important that genetic compatibility exists between the involved species to allow chromosome pairing, and it is especially fundamental that this pairing leads to real exchanges of chromatin between the donor chromosomes and the recipient ones; the success of hybridization depends on the recombination efficiency.

Another responsible factor for the success of breeding is the selection of genotypes with a high percentage of viable gametes (Techio et al. 2006). Pollen viability is considered a valuable tool for assessing the fertility of the male gamete and can be determined by using different methods: staining techniques, *in vitro* and *in vivo* germination tests, or analyzing final seed set (Abdelgadir et al. 2012). For staining techniques, different dyes such as acetic-carmine, Alexander’s solution, fluorescein diacetate, and Lugol reagent can be used. These chemical dyes react with cellular components of the mature pollen grain, indicating whether the pollen is viable or unviable (Lavinscky et al. 2017).

The observations of both meiotic behaviour and plant fertility can thus help reducing the time needed for producing new hybrid cultivars, since plants with meiotic irregularities and/or unviable pollen grains can be rejected for selection of more stable genotypes (Lavinscky et al. 2017).

The objective of this study was to develop backcross progenies from the HTL hybrid, and to analyse their meiotic behaviour and their fertility with the long-term objective of introgressing the improved fiber fineness trait from *G. longicalyx* into *G. hirsutum*.

## Material and methods

### Plant material

[(*Gossypium hirsutum* cv. C2 × *G. thurberi* G27)^2^ × *G. longicalyx* G17] (A_h_D_h_D_1_F_1_, 2n = 4x = 52) trispecies hybrid created by Konan et al. (2007) was backcrossed to *G. hirsutum* (cv. C2) to produce BC1 progenies. Crosses were achieved as follows: flowers of HTL trispecies hybrid were emasculated in the afternoon before anthesis and the stigma was covered by a small plastic sachet; pollen was applied to stigmas between 08:00 and 11:00 h the following morning. To avoid capsule shedding, a small piece of cotton wool containing a drop of the growth regulator solution (100 mg l^-1^ naphtoxyacetic acid + 50 mg l^-1^ gibberellic acid) recommended by Altman (1988) was applied on the ovary just after pollination.

Twenty seven BC1 seeds were hulled and cultivated *in vitro* on Murashige and Skoog medium (Murashige and Skoog 1962) because of their lack of germinative vigour. After a week of *in vitro* culture, seedlings were acclimated in a growth chamber (12 h of light, 55%–60% relative humidity and 28–26 °C day-night air temperatures). Twelve surviving BC1 adult plants were multiplied by grafting on *G. hirsutum* vigorous seedlings and cultivated in greenhouses for morphological observations, SSR marker analyses, pollen quality evaluation, meiosis analyses, selfings, backcrossings and, fiber fineness analyses. Plants were grown, in greenhouse, in 5 liter pots filled with a 3:2:1 (v:v:v) sterile mixture of compost, sand and peat. A dozen BC2 seeds belonging to the progeny of a randomly chosen BC1 plant were germinated on moist filter paper in a petri dish at 30 °C, for fast-growing root tips production in order to carry out genomic *in situ* hybridization.

### Analysis of meiosis

Cytological analyses on the plant material produced were performed on their pollen mother cells (PMC) at meiosis. Suitable flower buds of each plant were collected between 09:00 and 11:00, according to the weather conditions, and fixed in fresh Carnoy’s II fluid (glacial acetic acid 1: chloroform 3: and ethanol 6) for 72 hours at 4 °C. They were then stored at 4 °C in 70 % ethanol until their evaluation. To obtain meiotic plates, a few anthers were squashed in a drop of 1.5 % acetic-carmine solution on a microscope slide, debris were removed with fine forceps, and the slide was covered with a coverslip and heated a few seconds over a flame to improve chromosome staining. With pressure on the coverslip, pollen mother cells were flattened to spread out chromosomes. Observations were made with a Nikon Eclipse E800 photomicroscope (Nikon, Tokyo, Japan) under oil immersion. We concentrated our observations on metaphase I stage where chromosome arrangements (univalents, bivalents, and multivalents) were counted. But meiotic abnormalities such as laggard chromosomes at Anaphase I, Telophase I, Metaphase II, Anaphase II and final abnormal products of meiosis were considered as well.

### Evaluation of the plant fertility

To have an indication of pollen quality, about 300 pollen grains per plant were analyzed. Flowers were collected in the morning on the day of anthesis. Pollen grains were dipped in a drop of 1.5 % acetic-carmine solution on a slide for 30 minutes and were analysed under a stereomicroscope Nikon Eclipse E800 (Nikon, Tokyo, Japan). Only fully stained and large pollen grains were scored as viable and non-aborted. The quantity of viable pollen was estimated as the percentage of stained pollen.

The self-fertility of the BC1 plants was assessed by determining the average number of seeds obtained from 30 self-pollinated flowers of each BC1 genotype. The cross-fertility of the BC1 plants was assessed by counting the average number of BC2 seeds obtained per backcross.

### Fiber fineness analysis

For fiber fineness analysis, the fibers were combed and a tuft of parallel fibers was cut from the seed. Their free points were also cut and the median region was placed on a slide and covered with a cover glass. We let one or two drops of 18 % NaOH solution penetrate by capillarity into the fibers. The NaOH solution swells the fibers. The diameter of at least 100 fibers was then measured with the software NIS-Elements BR 2.30 using the Nikon Eclipse E800 microscope (Nikon, Tokyo, Japan) equipped with a digital JVC KY-F 58E camera (JVC, Yokohama, Japan). The ribbon width was determined by dividing the mean of the diameters measured by the 1.3 Summers coefficient (Roehrich 1947; Nacoulima et al. 2016). All the data collected were subjected to the analysis of variance (ANOVA) using the software Statistica 7.1 (Stat Soft France). The least significant difference (LSD) was used to establish differences between means at P = 0.05.

### DNA isolation and microsatellites marker analysis:

SSR marker analysis was achieved to check molecular segregation among the BC1 plants. Total genomic DNA was isolated from young fresh leaf tissues following the mixed alkyltrimethylammonium bromide (MATAB) method described by Lacape et al. (2003). SSR markers BNL 836, BNL 2662 and BNL 3279, developed at Brookhaven National Laboratory (BNL) and showing polymorphism between the three parental species were used. Clone sequences used for these primer definitions are available at http://ukcrop.net/perl/ace/search/cottonDB.

Polymerase chain reactions (PCR) were performed in 10 μL volume containing approximately 25 ng of template DNA, 0.6 U of Taq DNA Polymerase, 2.5 mM MgCl_2_, 1× Polymerase Buffer, 2 µM of each forward and reverse primers, and 0.2 mM of dNTPs mix. A PTC-200 thermal cycler (BioRad, Belgium) was used, with a PCR conditions consisting of an initial denaturation at 94 °C for 5 min, followed by 35 cycles of denaturation at 94 °C for 30 s, annealing at 55 °C for 1 min and extension at 72 °C for 1 min, with a final 72 °C extension for 8 min. Amplification products were separated on 6.0 % denaturing polyacrylamide gel and visualized by silver stain according to the protocol of Benbouza et al. (2006). The microsatellite bands were photo-documented and analyzed.

### Genomic *in situ* hybridization

To know whether the meiotic behaviour observed in the HTL trispecies hybrid and its BC1 derivatives allows recombination between *G. hirsutum* and *G. longicalyx* chromosomes, genomic *in situ* hybridizations (GISH) were performed using fast-growing root tips of twelve BC2 seeds belonging to the progeny of a randomly chosen BC1 plant.

DNA probe labelling

Total genomic DNAs were labelled by nick translation method with digoxigenin-11-dUTP (Roche, Switzerland) and biotin-14-dATP (Invitrogen life technologie, Carlsbad, USA) according respectively to the labelling protocol of the manufacturers. To reveal chromosomes or chromatin of *G. longicalyx* in the HLT hybrid and in BC2 derivative, Digoxigenin-11-dUTP was used to label total genomic DNA of *G. longicalyx* and biotin-14-dATP to label total genomic DNA of *G. hirsutum*.

Chromosome preparation

For mitotic metaphase chromosomes preparations, fast-growing root tips were collected in 0.04 % 8-hydroxyquinoline for 4 hours at room temperature (RT) and fixed for 48 h in a fresh fixative fluid (3:1 ethanol: acetic acid) at 4 °C. After washing in distilled water (2 × 10 min), treating in 0.25 N HCl (10 min), rinsing in distilled water (10 min) and treating in a 0,01M citrate buffer (10 min), root tips were digested in an enzyme solution (5 % cellulase Onozuka R-10, 1 % pectolyase Y-23 in citrate buffer) at 37 °C for 1 hour. The enzyme mix was removed by rinsing in distilled water for 10 min, and on a clean glass slide a single root tip was spread in one or two drops of fresh fixative (3:1 ethanol : acetic acid) using a fine-pointed forceps. Slides were stored at -20 °C until needed.

*In situ* hybridization

*In situ* hybridization was performed according to the protocol used by D’Hont et al. (1995). Slides were treated with RNAse A (1 µg/ml) at 37 °C for 1 hour, denatured for 2.5 min in 70 % deionized formamide in 2 × SSC (sodium saline citrate) at 70 °C, then dehydrated in an ethanol series of 70 %, 95 %, 100 % for five min at -20 °C, followed by air-drying. The hybridization mixture was 30 µl per slide and it contained 15 µl of 100 % deionized formamide, 3 µl of 20 × SSC, 6 µl of 50 % (w/v) dextran sulfate, l µl of 20 % SDS (sodium dodecyl sulphate) and 250 ng of each of the two DNA probes. The hybridization mixture was denatured for 10 min at 75 °C, chilled on ice for at least 5 min and added to the slide. Hybridization was performed in a humid chamber at 37 °C overnight.

Hybridization signal detection

After hybridization, to dissociate non-specific and imperfect hybrids, posthybridization stringent washes were performed successively in 2 × SSC, 0.5 × SSC, 0.1 × SSC, for 10 min each wash at 42 °C and in 2 × SSC for 10 min at 37 °C. Slides were afterwards incubated with 200 µl (per slide) of 5 % BSA-4SSC/Tween for 10 min at 37 °C. Both the BSA (bovine serum albumin) and the detergent Tween bind to the unoccupied sites, preventing subsequent non-specific binding of the antibodies. Biotin detection with Texas Red, digoxigenin with FITC (fluorescein isothiocyanate) and amplification were achieved as follows: Slides were incubated for 45 min at 37 °C three times; the first time with 50 µl of 5µg/ml Texas Red-avidin in TNB (100 mM Tris HCl (pH 7.5), 150 mM NaCl, 0.5 % Blocking reagent), the second time with 50 µl of a mixture of 12.5 µg/ml Biotinilated anti-avidin + 2 µg/ml anti-Dig FITC in TNB and lastly with 50 µl of a mixture of 5 µg/ml Texas Red-avidine + 5 µg/ml FITC-conjugated rabbit anti sheep in TNB. Each incubation was followed by two washes in TNT (100 mM Tris HCl (pH 7.5), 150 mM NaCl, 0.05 % Tween 20) at 37 °C for 5 min. The slides were wash for 1 min in 2 × SSC at 37 °C and dehydrated in an ethanol series of 70 %, 95 %, 100 % for 1 min at RT. Chromosome preparations were counterstained with DAPI (4,6-diamidino-2-phenylindole) in Vectashield. Slides were examined with an epifluorescence Nikon Eclipse E800 microscope (Nikon, Tokyo, Japan) using appropriate filters and a JVC KY-F 58E camera (JVC, Yokohama, Japan). Images were captured and processed with the softwares ArcSoft PhotoStudio 2000 4.3 and Adobe Photoshop 7.

## Results

### BC1 plants and their morphological observation

On 183 backcrosses achieved with the HTL hybrid and *G. hirsutum*, only 27 BC1 seeds (i.e. 0.15 seeds per cross) were obtained with generally one seed per boll. Thirteen of these seeds gave rise to viable plants. Among these plants, only twelve produced flower buds and could be submitted to cytogenetic analysis. An important segregation regarding morphological characters was observed among the BC1 plants. The plant heights ranged from 133 cm (BC1/10) to 241 cm (BC1/2), while the heights of *G. hirsutum* and the HTL were on average 150 and 274 cm respectively. The size of the BC1 plants leaves was variable but all of them were bigger than the leaves of *G. thurberi* and *G. longicalyx*, and close to those of *G. hirsutum*. The colour of the flowers was pale cream, light yellow or yellow. The BC1/10 plant had flowers with red spot at the base of the petals like *G. thurberi*. It was the sole BC1 plant that presented this trait. No pollen grains were noted on the anthers of the BC1/12 genotype. The anthers of this plant remained indehiscent even after the flower opened.

### Fiber fineness analysis

Table 1 shows the results of fiber fineness analysis. Among the three parental species, *G. longicalyx* had the finest fiber with 5.94 µm ribbon width against 17.765 and 15.769 µm for *G. hirsutum* and *G. thurberi* respectively. The ribbon width of the HTL trispecies hybrid was 12.649 µm, while it ranged from 13.039 to 16.276 µm for the BC1 plants. Among these BC1 plants, BC1/4, BC1/3, BC1/11, and BC1/6 had the lowest ribbon width (13.039–13.416 µm) while BC1/1 and BC1/2 had the highest values (16.235 and 16.276 µm respectively).

**Table 1.** Ribbon width of parental species and the BC1 progenies of the HTL trispecies hybrid.

Genotype	Number of fibers analysed	Ribbon widh (µm) ± standard deviation	LSD grouping
*G. hirsutum* (cv. C2)	107	17.765 ± 0.130	H
*G. thurberi*	107	15.769 ± 0.130	I
*G. longicalyx*	113	5.940 ± 0.126	A
HTL hybrid	120	12.649 ± 0.123	B
HTL BC1/1	112	16.235 ± 0.127	J
HTL BC1/2	111	16.276 ± 0.128	J
HTL BC1/3	122	13.414 ± 0.122	D
HTL BC1/4	110	13.039 ± 0.128	C
HTL BC1/5	108	15.347 ± 0.129	H
HTL BC1/6	124	13.336 ± 0.121	CD
HTL BC1/7	110	14.822 ± 0.128	G
HTL BC1/8	115	14.457 ± 0.125	F
HTL BC1/9	111	14.358 ± 0.128	EF
HTL BC1/10	111	14.081 ± 0.128	E
HTL BC1/11	114	13.41 ± 0.126	D
HTL BC1/12	111	14.327 ± 0.128	EF

### SSR marker analysis

**Figure 1.**
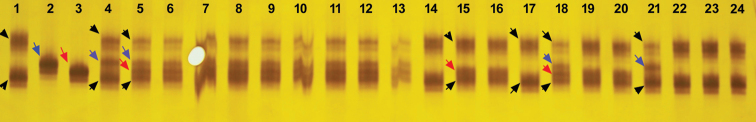
Polyacrylamide gel with BNL3279 SSR marker presenting allele segregations in the parental species, the hexaploid, the HTL tri-species hybrids and the BC1 plants. **1***G. hirsutum***2***G. thurberi***3***G. longicalyx***4** the hexaploid (*G. hirsutum* × *G. thurberi*)^2^**5–13** HTL tri-species hybrid plants (*G. hirsutum* x *G. thurberi*)^2^ × *G. longicalyx***14–24** BC1 plants (HTL x *G.*hirsutum). Black arrow: allele of *G. hirsutum*, Blue arrow: allele of *G. thurberi*, Red arrow: allele of *G. longicalyx*.

All the SSR markers used revealed polymorphism between the parental species. They also showed segregation of the diploid species alleles among the BC1 plants (Table 2). For each of the three primers, seven BC1 plants (BC1/1, BC1/4, BC1/9, BC1/10, BC1/11 and BC1/12) were homozygous exhibiting only *G. hirsutum* alleles, while three BC1 plants (BC1/2, BC1/3 and BC1/6) were heterozygous showing alleles from *G. hirsutum* and *G. longicalyx*. For BNL327 and BNL2662, the BC1/5 plant was heterozygous with alleles from the three parental species and BC1/8 was heterozygous with alleles from *G. hirsutum* and *G. thurberi*. For BNL836, these two plants were respectively heterozygous (with alleles from *G. hirsutum* and *G. longicalyx*) and homozygous (with alleles from *G. hirsutum*). Figure 1 gives an example of the alleles segregation observed with BNL3279.

**Table 2.** Distribution of homozygous (hh) and heterozygous (hl, ht or hlt) for SSR marker BNL832, BNL3279 and BNL2662 in the BC1 progeny (h: allele of *G. hirsutum*; l: allele of *G. longicalyx*; t: allele of *G. thurberi*).

Genotype	BNL 836	BNL3279	BNL2662
HTL BC1/1	hh	hh	hh
HTL BC1/2	hl	hl	hl
HTL BC1/3	hl	hl	hl
HTL BC1/4	hh	hh	hh
HTL BC1/5	hl	hlt	hlt
HTL BC1/6	hl	hl	hl
HTL BC1/7	hh	hl	hl
HTL BC1/8	hh	ht	ht
HTL BC1/9	hh	hh	hh
HTL BC1/10	hh	hh	hh
HTL BC1/11	hh	hh	hh
HTL BC1/12	hh	hh	hh

### Plant fertility

**Figure 2.**
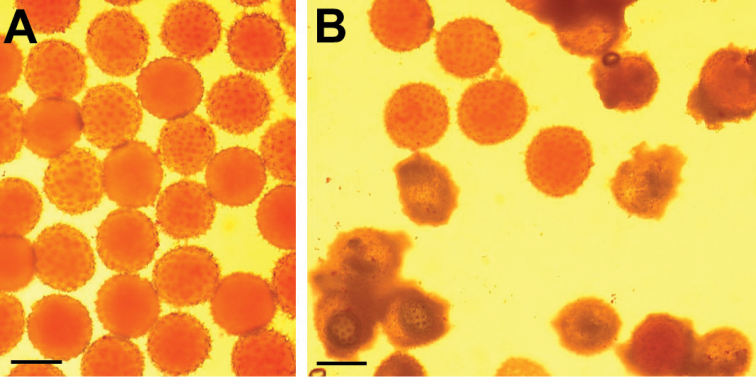
Cotton pollen grain viability revealed by acetic-carmine staining. **A***G. hirsutum* viable pollen grains with good colour and uniform size **B** mixture of viable and non viable pollen grains of a [(*G. hirsutum* × *G. thurberi*)^2^ × *G. longicalyx*] backcross-1 plant. Scale bars: 100 μm.

HTL BC1 plants presented great variations among them regarding pollen grain shape, size and stainability, unlike the pollen of the HTL parental species (*G. hirsutum*, *G. thurberi* and *G. longicalyx*) which had uniform size and were easily stainable with acetic-carmine (Fig. 2). The mean proportion of stainable pollen grains approached 100 % for the parental species, and ranged from 4.67 to 32.10 % for the BC1 plants (Table 3). The results of the self-pollinations are presented in Table 3. In the parental species, all the self-pollinated flowers gave mature bolls with a large number of well formed seeds. No mature capsule could be obtained by self-pollination of a large part of the HTL BC1 progeny (BC1/2, BC1/4, BC1/6, BC1/8, BC1/9, BC1/11 and BC1/12). A few mature capsules containing 0 to 12 seeds were produced by five BC1 plants (BC1/1, BC1/3, BC1/5, BC1/7 and BC1/10).

**Table 3.** Self-fertility of the first backcrossing (BC1) progeny of [(*G. hirsutum* × *G. thurberi*)^2^ × *G. longicalyx*] trispecies hybrid (HTL) and its parental species.

Genotype	Total number of examined pollen grain (% of stainable pollen grain)	No of self-fertilized flowers	No of aborted capsules	No of capsules harvested		No of seeds harvested	Average number of seeds per self-fertilized flower
				No of capsules without seeds	No of capsules containing seeds		
*G. hirsutum* (cv. C2)	200 (100.00)	30	0	0	30	1020	34
*G. thurberi*	131 (100.00)	30	0	0	30	450	15
*G. longicalyx*	200 (100.00)	30	0	0	30	180	6
HTL BC1/1	362 (15.19)	30	26	4	0	0	0
HTL BC1/2	321 (4.67)	30	30	0	0	0	0
HTL BC1/3	348 (14.37)	30	28	1	1	1	0.03
HTL BC1/4	383 (22.45)	30	30	0	0	0	0
HTL BC1/5	416 (11.78)	30	26	1	3	6	0.2
HTL BC1/6	352 (23.39)	30	30	0	0	0	0
HTL BC1/7	405 (32.10)	30	27	0	3	12	0.4
HTL BC1/8	383 (13.84)	30	30	0	0	0	0
HTL BC1/9	267 (9.36)	30	30	0	0	0	0
HTL BC1/10	332 (9.94)	30	28	0	2	9	0.3
HTL BC1/11	335 (10.45)	30	30	0	0	0	0
HTL BC1/12	Indehiscent anthers	30	30	0	0	0	0

Table 4 shows the results obtained for the backcrossing to *G. hirsutum* of the different BC1 plants. All the BC1 plants gave seeds by backcrossing. The number of BC2 seeds produced per BC1 plants ranged from 10 to 364 seeds with an average of 0.44 to 6.50 seeds per cross. The total number of BC2 seeds obtained after pollination of 508 flowers was 1215 seeds. BC1/10, BC1/5 and BC1/7 gave the highest mean numbers of seeds per backcross (6.5, 5.16 and 5.09 respectively), while BC1/9, BC1/12, BC1/1, BC1/11, BC1/6 and BC1/4 gave mean numbers of seeds inferior to 1 (0.44, 0.48, 0.55, 0.57, 0.96 and 0.98 respectively).

**Table 4.** Cross-fertility of the HTL BC1 plants (as females) with *G. hirsutum*.

Backrcrosses	No of pollinated flowers	No of capsules harvested		No of seeds harvested	Mean number of seeds per backcross
		No of capsules without seeds	No of capsules containing seeds		
HTL BC1/1 × *G. hirsutum*	20	10	10	11	0.55
HTL BC1/2 × *G. hirsutum*	33	0	33	92	2.79
HTL BC1/3 × *G. hirsutum*	17	1	16	36	2.12
HTL BC1/4 × *G. hirsutum*	43	20	23	42	0.98
HTL BC1/5 × *G. hirsutum*	37	0	37	191	5.16
HTL BC1/6 × *G. hirsutum*	23	13	10	22	0.96
HTL BC1/7 × *G. hirsutum*	43	0	43	219	5.09
HTL BC1/8 × *G. hirsutum*	74	8	66	157	2.12
HTL BC1/9 × *G. hirsutum*	75	53	22	33	0.44
HTL BC1/10 × *G. hirsutum*	56	7	49	364	6.5
HTL BC1/11 × *G. hirsutum*	66	39	27	38	0.57
HTL BC1/12 × *G. hirsutum*	21	12	9	10	0.48
**Total**	**508**	**163**	**345**	**1215**	**2.39**

### Meiosis of BC1 plants

**Figure 3.**
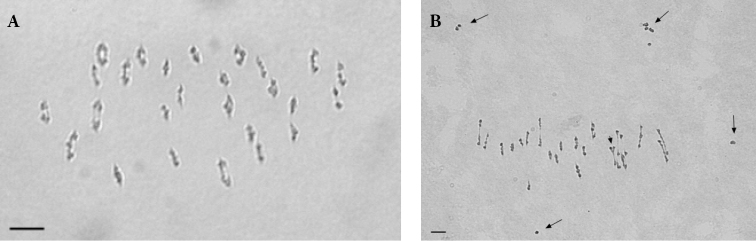
Meiotic metaphase I plates in *G. hirsutum* and in the HTL BC1/4 plant. **A** Meiotic metaphase I cell showing 26 bivalents in control *G. hirsutum***B** meiotic metaphase I cell showing 8 univalents (long arrows), 20 bivalents and 1 quadrivalent (short arrow) in HTL BC1/4. Scale bars: 5 μm.

Meiosis studies were performed on the HTL BC1 progeny, and also on *G. hirsutum* as control. With the exception of the HTL BC1/12 plant which had 54 chromosomes, all the analyzed HTL BC1 plants had 52 chromosomes. At metaphase I, chromosomes of *G. hirsutum* paired perfectly with 26 bivalents (Fig. 3A). The meiosis of *G. hirsutum* was regular, stable and normal tetrads with four normal cells were observed as final products. Chromosome associations at metaphase I in the HTL BC1 progeny were variable, with usually a mixture of univalents and bivalents (Fig. 3B) and sometimes multivalents. Cytological data concerning these genotypes are summarized in Table 5. In the BC1 plants, the chromosome associations varied as follows: (2.5 to 11.5) I + (17 to 22) II + (0.31 to 1.93) III + (0.09 to 1.93) IV + (0 to 0.07) V + (0 to 0.14) VI. On an average, the number of paired chromosomes observed in BC1 plants varied from 40.50 to 49.50. The plants BC1/10 and BC1/7 gave the lowest mean numbers of univalents (2.5 and 3.75 univalents respectively). The highest number of univalents was found with BC1/11 (11.50 on average).

**Table 5.** Meiotic pairing in *G. hirsutum* and in the HTL BC1 progeny.

Genotype	No of analyzed c ells	Chromosome configuration						Chromosome Number	Average No of chromosomes paired
		I	II	III	IV	V	VI		
*G. hirsutum*	10		26					52	52
HTL BC1/1	30	6.90 (1–12)	21.07 (17–24)	0.53 (0–3)	0.33 (0–2)	0.03 (0–1)		52	45.23
HTL BC1/2	32	9.37 (4–16)	20.66 (18–24)	0.31 (0–2)	0.09 (0–1)			52	46.62
HTL BC1/3	30	5.23 (0–12)	21.93 (16–26)	0.43 (0–2)	0.43 (0–2)			52	46.90
HTL BC1/4	30	7.73 (4–12)	20.67 (16–24)	0.37 (0–2)	0.40 (0–2)	0.03 (0–1)		52	42.20
HTL BC1/5	12	4.75 (2–10)	21 (19–24)	0.75 (0–3)	0.75 (0–2)			52	47.25
HTL BC1/6	33	5.85 (2–10)	21.67 (17–24)	0.45 (0–4)	0.36 (0–2)			52	46.15
HTL BC1/7	30	3.83 (0–8)	22.8 (19–25)	0.37 (0–3)	0.33 (0–2)			52	48.03
HTL BC1/8	37	8.63 (4–14)	18.94 (13–23)	0.51 (0–4)	0.97 (0–4)			52	43.32
HTL BC1/9	21	10.38 (5–15)	18.90 (15–22)	1.14 (0–3)	0.09 (0–2)			52	41.62
HTL BC1/10	14	2.5 (0–5)	22 (0–2)	1.07 (0–2)	0.57 (0–1)			52	49.50
HTL BC1/11	32	11.50 (7–17)	17.81 (10–22)	1 (0–4)	0.47 (0–3)			52	40.50
HTL BC1/12	14	5.28 (0–8)	17 (14–20)	1.93 (0–6)	1.93 (0–4)	0.07 (0–1)	0.14 (0–1)	54	48.71

**Figure 4.**
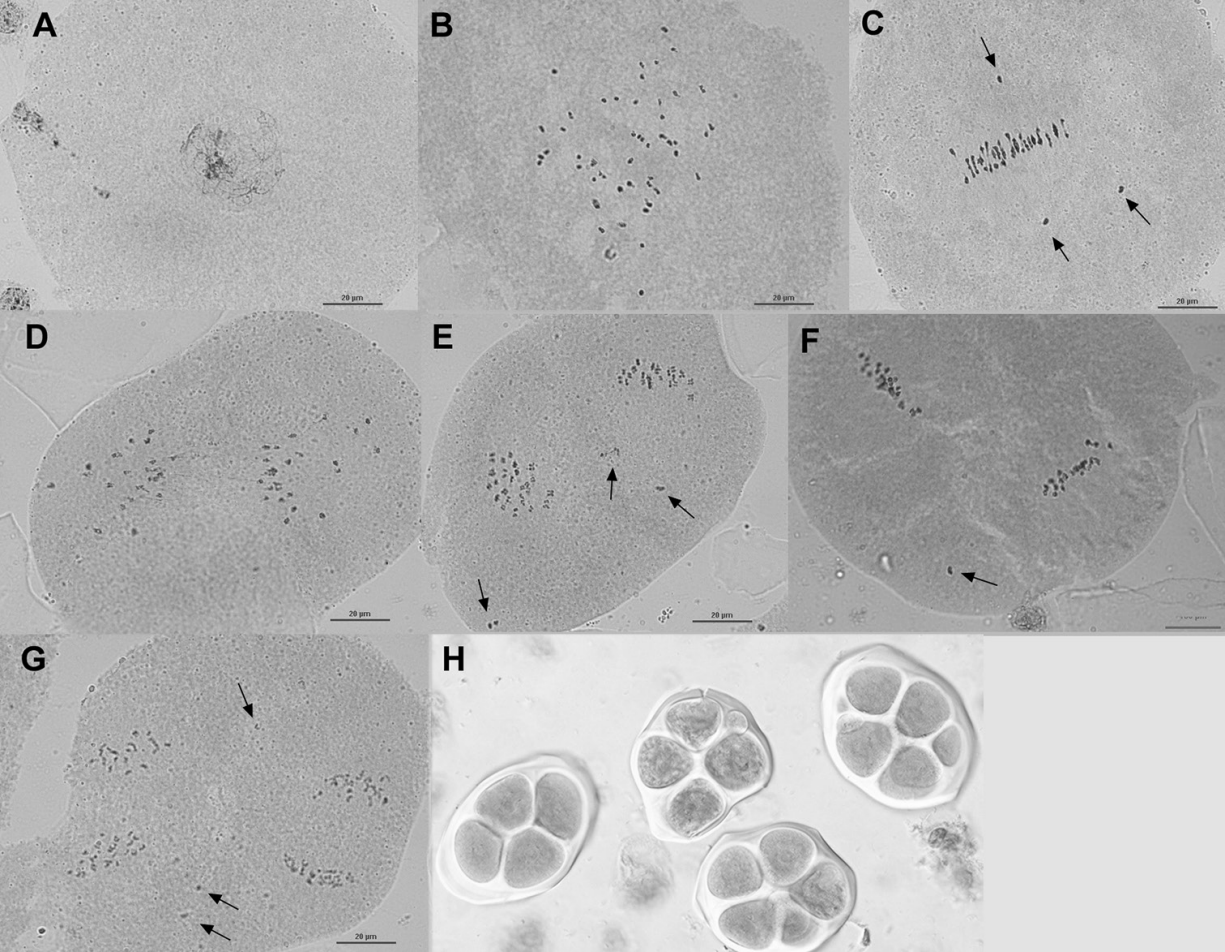
Meiotic aspect with abnormalities in HTL BC1 plants. **A** Leptotene **B** diakinesis **C** metaphase I with univalent chromosomes in early ascension (arrow) **D** anaphase I **E** telophase I with the presence of laggard chromosomes (arrows) **F** metaphase II with laggard chromosomes (arrows) **G** anaphase II with presence of laggard chromosome (arrow) **H** microsporocytes with a mixture of tetrad and polyads with micronuclei. Scale bars: 20 μm.

Unlike observations made in *G. hirsutum*, PMC meiosis in the BC1 progeny was mostly abnormal (Fig. 4) and asynchronous with different meiosis phases in the same flower bud. In these plants, from metaphase I onwards (Fig. 4C) meiosis showed abnormalities. The most common meiotic abnormality found was irregular chromosome segregation, characterized by precocious chromosome ascension and laggards. This irregular chromosome segregation affected all meiotic phases, generating genetically unbalanced microspores. Irrespective of the BC1 plants the meiotic behaviour was mostly the same, with univalents showing precocious ascension at metaphase I (Fig. 4C) or remaining as laggards at anaphase I, telophase I (Fig. 4E), metaphase II (Fig. 4F) and anaphase II (Fig. 4G). Chromosomes did not have the same rhythm in cell division and laggards were engulfed by extra nuclei so that at the end of the second division, meiotic products were mostly represented by polyads (more than 4 cells), generally with microspores of different sizes. In the BC1 plants, the final products of meiosis were, thus, completely abnormal, with a predominance of polyads even if some sporadic normal tetrads were observed (Fig. 4H).

### GISH analysis

**Figure 5.**
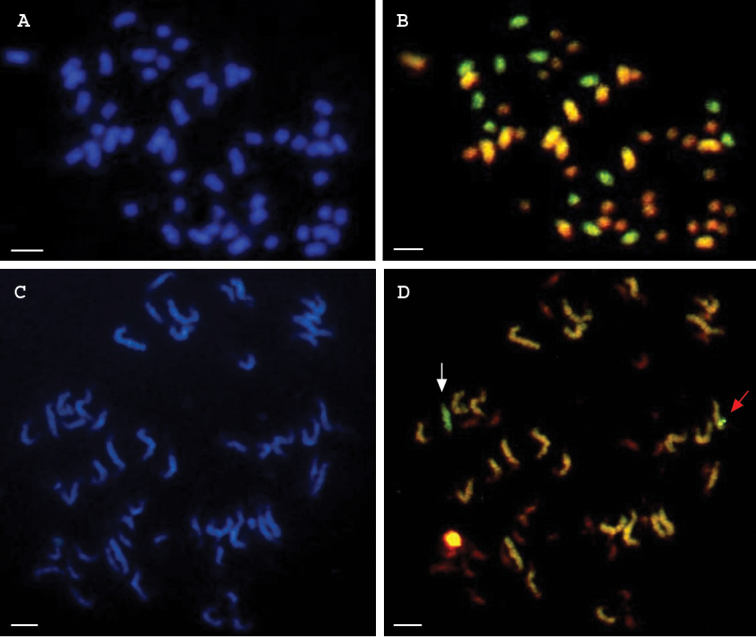
Genomic *in situ* hybridization on mitotic metaphase chromosomes of the HTL trispecies hybrid [(*G. hirsutum* × *G. thurberi*)^2^ × *G. longicalyx*)] and a BC2 progeny. **A** Mitotic metaphase showing 52 chromosomes of the HTL hybrid revealed by counterstaining with DAPI **B** mitotic metaphase showing in the HTL hybrid 13 green chromosomes from *G. longicalyx*, 13 yellow-orange chromosomes from the A-subgenome of *G. hirsutum* and 26 red chromosomes from *G. thurberi* and the D-subgenome of *G. hirsutum* revealed after the superimposition of FITC detection and Texas Red detection **C** mitotic metaphase showing 52 chromosomes in a HTL BC2 revealed by counterstaining with DAPI **D** mitotic metaphase in a HTL BC2 showing an entire chromosome of *G. longicalyx* (white arrow) and an intergenomic recombination (red arrow) showing movement of *G. longicalyx* chromatin into a chromosome of the A-subgenome of *G. hirsutum*. Scale bars: 5 μm.

Figure 5 shows results of GISH analysis. For the HTL hybrid and the BC2 progeny, 52 chromosomes appeared in blue when they were counterstained with DAPI (Fig. 5A, C). When FITC detection and Texas Red detection were superimposed, three populations of chromosomes are differentiated for the HTL trispecies hybrid (Fig. 5B). Thirteen chromosomes appeared green and were those from *G. longicalyx*; thirteen large chromosomes appeared yellow-orange and were those from *G. hirsutum* A-subgenome, twenty six small chromosomes appeared red and were those from *G. hirsutum* D-subgenome and *G. thurberi* D1 genome since the D genome is comprised of the smallest chromosomes (Phillips and Strickland 1966; Konan et al. 2009; Li et al. 2016). For the Backcross-2 progenies, GISH revealed presence of entire chromosomes of *G. longicalyx* as well as recombinant chromosomes (Fig. 5D).

## Discussion

Compared with the homologous chromosome pairing in the parental *G. hirsutum* species which showed only bivalents, the meiotic behaviour of HTL BC1 progeny was abnormal. In these plants, a disturbed meiosis was observed and, at metaphase 1, chromosomes paired imperfectly and gave, in addition to bivalents, some univalents and multivalents. The same abnormalities were observed by Konan et al. (2007) with the HTL trispecies hybrid. Moreover, *G. hirsutum* gave normal tetrad and 100% pollen fertility unlike the BC1 plants. The normal and harmonious course of meiosis in pollen mother cells of *G. hirsutum* with regular bivalent formation and normal tetrad production after cytokinesis ensured 100 % pollen viability; whereas the abnormalities observed in the meiosis of the BC1 plants caused the formation of sterile gametes and low percentages of pollen viability. These results indicate a link between meiotic behaviour and pollen fertility. They suggest that the irregular chromosome pairing observed in the BC1 plants is one of the main reasons of the problem of fertility observed. Usually in plant species with normal genomes, homologous chromosomes tend to have high matching rate unlike the homoeologous chromosomes in hybrid plants derived from interspecific crosses (Kaur and Singhal 2019). Actually, an essential event in meiosis is the chromosome pairing, not only for the occurrence of recombination but also for the correct chromosome segregation. Working on meiotic irregularities and pollen viability in the genus *Passiflora* Linnaeus, 1753, Souza et al. (2003) showed that when an error occurs in the chromosome pairing, the segregation can happen in an unbalanced way and gametes can receive an unbalanced number of chromosomes, leading to decrease of viable gametes. This is in accordance with several other authors (Kammacher 1966, Shuijin and Biling 1993, Zanders et al. 2014, Potapova and Gorbsky 2017, Wang et al. 2017) who found that the irregular chromosome pairing could cause unequal meiotic division and unbalanced chromosome segregation, leading to incomplete or abnormal chromosome sets (i.e. aneuploidy) after cytokinesis and causing sterile gametes.

Konan et al. (2007) observed the same meiotic abnormalities with the HTL trispecies hybrid (14.13 I + 15.10 II + 1.03 III + 0.9 IV + 0.03 V + 0.13 VI). But there was globally, greater chromosome pairing in the BC1 plants than in the trispecies hybrid (40.50 to 49.50 instead of 37.85 paired chromosomes in HTL), supporting their relative higher pollen fertility. In general, progression toward increasing frequencies of plants that form 26 bivalents at metaphase I were observed in the advanced backcross generations of other cotton trispecies hybrids exploited in breeding programs (Vroh et al. 1999, Mergeai 2006). This was also the case for the HTL BC1 progeny. This observation indicates that backcrossing has promoted cytological stability. He et al. (2017) made the same observation in their works on chromosome pairing in backcross progeny of the hybrid *Triticum aestivum* Linnaeus, 1753 and *Elytrigia elongata* (Host ex P. Beauvois, 1812) Nevski, 1933.

Four BC1 genotypes were able to produce a few seeds through self-pollination. These plants which produced also the highest number of BC2 seeds per backcross were characterized by a better meiotic stability. Their relative lower univalent rate and higher paired chromosome rate could explain their higher self- and cross-fertility. Globally, the number of BC2 seeds produced per backcross was negatively correlated with the average number of univalents at Metaphase I (R = -0.67, P < 0.05) indicating a deleterious role of unpaired chromosomes on the fertility of the hybrid plants. This is in accordance with Bikchurina et al. (2018) who reported that univalents impair pollen fertility and seed production.

However, irregular meiotic behaviour cannot be the sole explanation of the observed sterility problems. Indeed, some of the HTL BC1 plants presenting a few univalents were totally self-sterile, indicating that additional factors may reduce the actual gamete viability of the BC1 plants. The same observation was earlier made by Brown and Menzel (1950) in the [(*G. hirsutum* × *G. arboreum*)^2^ × *G. harknessii* Brandegee, 1889] and [(*G. hirsutum* × *G. herbaceum*)^2^ × *G. harknessii*] trispecies hybrids and by Kammacher (1956) in the allotetraploid hybrid (*G. arboreum* × *G. thurberi*)^2^. These three hybrids characterised by relatively good chromosome pairing rates (respectively 24.6, 22.36 and 22.14 bivalents) were also self-sterile. Actually, each species has a balanced complex of many genes and a structural organization of the chromosomes that regulates pairing and ensures the genetic stability of the species (Mursal and Endrizzi 1976, Jeridi et al. 2011). Hybridization between species can destroy this stability. Homoelogous chromosomes of two close species can differ by several little chromosomal rearrangements appeared during genome differentiation (Jeridi et al. 2011). Even if hybrids between such species do not show irregular chromosome configuration at meiosis owing to their apparent homology, the genetic stability is however disturbed and the hybrids can be sterile (Jeridi et al. 2011). Sterility of plants with regular meiosis has been also associated with disturbances of tapetal development and degeneration (Narkhede et al. 1967, Papini et al. 1999, Wang et al. 2015). Tapetum is the innermost of the four sporophytic layers of the anther wall that plays an important role in the male fertility of pollen grains. It comes into direct contact with the developing male gametophyte and contains all the nutrients for microspore development and maturation, such as callose, sporopollenin and proteins (Wang et al. 2015). Studies have proven that tapetal tissue has a secretory role, providing nutrients required for microspore and pollen grain development, and defects in tapetal tissue can lead to pollen abortion (Chiavarin et al. 2000, Wang et al. 2015). While studying the stage of pollen grain degeneration of male sterile plants in sorghum, wheat, tomato, sugar-beets, maize etc. earlier authors (Narkhede et al. 1967) have already reported that meiosis in these male sterile plants appeared to be normal, but after the pollen grains were partially formed they aborted and the anthers lacked viable pollen prior to dehiscence. According to these authors even if the meiosis was found to be normal in the male sterile plants, the pollen grains deteriorated after their formation because the tapetum in anthers of such plants did not deteriorate and release the food material necessary for the normal development of the pollen grains. It was stated that there was a possible association of the nonviable character of pollen grains with the nutritive role of tapetum in male sterile plants. Recently, there have been a large number of reports that confirmed this statement (Ma et al. 2015, Wang et al. 2015, Li et al. 2017, Zheng et al. 2019).

Although only four BC1 plants produced a few seeds through self-pollination, all the BC1 plants gave seeds by backcrossing when they were used as female. This result indicates that instead of the male sterility presented by most of BC1 plants, the potential for female reproduction remains. Konan et al. (2007) made the same observation with the HTL trispecies hybrid.

Evaluation of fiber fineness showed that *G. longicalyx* had the finest fiber among the parental species, and the HTL trispecies hybrid and some of its BC1 progenies gave finer fiber than the main cultivated cotton *G. hirsutum*. This result confirms that *G. longicalyx* is a good donor for fiber fineness. The plants BC1/3, BC1/4, BC1/6, and BC1/11 which gave the lowest ribbon width are interesting genetic stocks which can be selected for further improvement of this trait in a breeding program. However, as one of the main success factors of breeding is the selection of genotypes with a high percentage of viable gametes (Lavinscky et al. 2017), the BC1/10 stock which was the most stable plant (with the lowest univalent number, the highest paired chromosome number, the highest cross fertility, and among the best self-fertile plants) and had a fairly good fiber fineness can be selected as well.

SSR marker analysis revealed segregation of diploid alleles among the BC1 plants indicating the differential presence or absence of the diploid species chromosomes and/or chromosome recombinations. This allele segregation supports the segregation observed in BC1 plants regarding fiber fineness trait and the other morphological traits. For the success of an interspecific breeding program, homoeologous recombinations are crucial. Interspecific hybridization finds its justification in the possibility for genetic material exchanges between the genome of the different target species. The degree of homology between the parental genomes is fundamental for the occurrence of intergenomic recombination in successive backcross progenies. In general, chromosomes of closely related genomes tend to pair more often than chromosomes of genomes that are more distantly related (Kimber and Yen 1990, He et al. 2017). The occurrence of chromosome pairing provides a basis for recombination. According to some authors (Brown and Menzel 1952; Endrizzi 1957; Phillips and Strickland 1966; Konan et al. 2009) the D-subgenome of *G. hirsutum* retains sufficient chromosome homology with the D_1_ genome of *G. thurberi*, and the F_1_ genome of *G. longicalyx* is more closely related to the *G. hirsutum* A-subgenome. Therefore, in the hybrid plants, D_1_ chromosomes of *G. thurberi* should mostly pair with the chromosomes of *G. hirsutum* D-subgenome, while F_1_ chromosomes of the donor species (*G. longicalyx*) should mostly pair with the chromosomes of *G. hirsutum* A-subgenome. The formation of a high number of bivalents and some multivalents as it was observed in these hybrids is an indication that genomic exchanges can occur during the meiosis between wild genome chromosomes and the chromosomes of *G. hirsutum* subgenomes. GISH revealed a clear distinction of the *G. hirsutum* and *G. longicalyx* chromosomes in the trispecific hybrid, showing that it is possible to differentiate *G. longicalyx* and *G. hirsutum* chromatin. For the backcross derivative progenies, GISH revealed presence of entire chromosomes of *G. longicalyx* as well as recombinant chromosomes, indicating that segregation of *G. longicalyx* chromosomes and introgression of this species chromatin into *G. hirsutum* occur. These results show that it is possible to introgress chromosome segments of *G. longicalyx* into *G. hirsutum*. Segregation observed in the BC1 plants regarding morphological characters could hence be attributed to the differential presence or absence of the diploid species chromosomes and to homoelogous chromosome recombination as suggested by SSR marker analysis. The current results suggest that the development of balanced upland cotton varieties integrating *G. longicalyx* genetic material and exhibiting good fiber fineness could be feasible.

## Conclusion

Cotton fibers sustain one of the world’s largest industries, the textile industry, for wearing apparel, home furnishings, and medical supplies. Further improvement of cotton fiber quality is much desired. Most of the BC1 plants studied have presented an increased number of paired chromosomes compared to the parental HTL trispecies hybrid. Moreover, all of them could produce seeds through backcrossing and some were self-fertile. The plant material is gaining stability. Some BC1 plants exhibited interesting fiber fineness and recombination is possible between the donor species chromosomes and *G. hirsutum* chromosomes. These results are promising for the introgression of the improved fiber fineness trait from *G. longicalyx* into upland cotton.
